# Mammographic density and epithelial histopathologic markers

**DOI:** 10.1186/1471-2407-9-182

**Published:** 2009-06-13

**Authors:** Martijn Verheus, Gertraud Maskarinec, Eva Erber, Jana S Steude, Jeffrey Killeen, Brenda Y Hernandez, J Mark Cline

**Affiliations:** 1 Cancer Research Center, University of Hawaii, Honolulu, Hawaii, USA; 2 Department of Pathology, Section on Comparative Medicine, Wake Forest University School of Medicine, Winston-Salem, North Carolina, USA; 3 Kapiolani Medical Center for Women and Children, Honolulu, Hawaii, USA

## Abstract

**Background:**

We explored the association of mammographic density, a breast cancer risk factor, with hormonal and proliferation markers in benign tissue from tumor blocks of pre-and postmenopausal breast cancer cases.

**Methods:**

Breast cancer cases were recruited from a case-control study on breast density. Mammographic density was assessed on digitized prediagnostic mammograms using a computer-assisted method. For 279 participants of the original study, we obtained tumor blocks and prepared tissue microarrays (TMA), but benign tissue cores were only available for 159 women. The TMAs were immunostained for estrogen receptor alpha (ERα) and beta (ERβ), progesterone receptor (PR), HER2/neu, Ki-67, and Proliferating Cell Nuclear Antigen (PCNA). We applied general linear models to compute breast density according to marker expression.

**Results:**

A substantial proportion of the samples were in the low or no staining categories. None of the results was statistically significant, but women with PR and ERβ staining had 3.4% and 2.4% higher percent density. The respective values for Caucasians were 5.7% and 11.6% but less in Japanese women (3.5% and -1.1%). Percent density was 3.4% higher in women with any Ki-67 staining and 2.2% in those with positive PCNA staining.

**Conclusion:**

This study detected little evidence for an association between mammographic density and expression of steroid receptors and proliferation markers in breast tissue, but it illustrated the problems of locating tumor blocks and benign breast tissue samples for epidemiologic research. Given the suggestive findings, future studies examining estrogen effects in tissue, cell proliferation, and density in the breast may be informative.

## Background

Although a vast body of literature describes a positive association between mammographic density and breast cancer risk with an estimated relative risk of 4 or higher for women in the highest as compared to the lowest density category [1], not much is known about the underlying histopathology of breast density [2]. Such knowledge may contribute to breast cancer prevention because it may improve our understanding of the relation between density and breast cancer risk as well as the potential for risk prediction and modification. The two types of tissue that give rise to radiologically dense breasts are epithelium and stroma forming the microenvironment of epithelial cells which constitute less than 5% of breast tissue [3,4]. The main component of stromal tissue is collagen [5]. It was hypothesized that the extent of mammographic density is proportional to the amount of breast epithelium and that the higher breast cancer risk associated with breast density is due to a larger number of glandular cells at risk for malignant transformation [6,7]. This idea is supported by findings of an association between the proliferation of stroma, epithelium, or both with breast density in subjects with breast abnormalities [8,9]. Unfortunately, research in healthy women is limited to forensic studies [10,11] and one study of breast reduction samples [5]. In breast cancer patients, increased amounts of collagen were associated with breast density in several reports [5,9,11,12], while the results on cell proliferation were mixed [5,13,14].

As risk factors that induce cell proliferation [15,16], endogenous sex steroids and hormone therapy (HT) are associated with higher breast cancer risk [17,18]. Whereas HT, in particular estrogen plus progestogen therapy, increases mammographic density [19], the relation between endogenous sex steroids and mammographic density is less clear. One study observed an association with endogenous estrogens [20] but others did not [21-23]. As breast tissue levels are partly determined by estrogen production in adipose tissue, breast size as marker for adipose tissue surrounding the epithelial cells may possibly be a better marker for tissue levels than circulating estrogen levels. Endogenous progesterone was found to be related to mammographic density in one report among premenopausal women [24]. The biological activities of endogenous and exogenous estrogens on breast tissue are mediated by nuclear estrogen receptors (ER) α and β. Differential effects of ERα and ERβ are of interest because ERβ appears to be more antiproliferative while ERα has proliferative activity [25,26]. Progesterone, an ovarian steroidal hormone, acts through its specific receptor (PR) [27,28]; PR expression has been shown to be a sensitive indicator of estrogenic effects in cells [29].

To understand how hormone receptors and cell proliferation are related to breast density, this study examined the expression of ERα, ERβ, and PR as well as HER2, Ki-67, and Proliferating Cell Nuclear Antigen (PCNA) [30], in relation to mammographic density among breast cancer patients with Caucasian, Japanese, and Hawaiian ethnicity. We are convinced that these associations are best studied in benign breast tissue and, thus, restricted this analysis to breast cancer patients for whom benign tissue samples placed on tissue microarrays (TMA) were available. The relation between mammographic appearance of the breast and marker expression in tumor tissue is a separate issue that needs further study [31].

## Methods

### Study population

The study was approved by the Institutional Review Boards of the University of Hawaii and Wake Forest University; all subjects provided informed consent in writing. We recruited subjects for the TMA study from 607 breast cancer cases who had participated in the Multiethnic Cohort (MEC) [32] and a nested case-control (NCC) study of mammographic densities [33]. Of these, 177 women were excluded because their tumor blocks were not available from the Hawaii Tumor Registry (HTR). Recruitment letters and questionnaires were mailed to the remaining 430 subjects; 323 (75%) women returned the consent forms. Another 12 women were deceased but linked to the HTR and could, thus, be included in the study. For 279 out of these 335 subjects, pathologic blocks from breast cancer surgery were located and used to create TMAs; no tissue from prior benign biopsies was available. At entry into the MEC, all participants had completed a questionnaire that inquired about demographics, reproductive behavior, anthropometric measures, and family history of breast cancer [32]. As part of the NCC, women completed an additional one-page breast health questionnaire that asked about previous breast surgery, menopausal status, mammography history, and HT use [33].

### Tumor microarrays

TMAs were prepared according to standard procedures [34,35]. In brief, a surgical pathologist (JK) identified blocks from a given patient with sufficient tissue. For each of these blocks a single hematoxylin and eosin (H&E) slide was prepared on which the same pathologist marked representative areas of malignant and benign tissue. The H&E slide was aligned with the corresponding "donor" block and a 0.6 mm cylindrical tissue specimen was taken from the selected area and transferred to a "recipient" paraffin block using a tissue-arraying instrument (Beecher Instruments, Sun Prairie, WI). When available, four malignant cores and four benign cores per patient were placed in one of six blocks. Out of the 2,232 cores to be placed (four malignant and four benign samples for 279 women), tissue was insufficient for 12% of malignant and 29% of benign specimens resulting in 1,773 tissue samples (79.4%) for analysis. At least one benign or malignant core was available for 268 women. Several 5 μm sections were mailed to Wake Forest University for immunohistochemical staining.

### Immunohistochemistry and pathologic evaluation

The TMAs were stained for the following markers: ERα, ERβ, PR, and PCNA (Clones 6F11, EMR02, 1A6, and PC10, respectively; all from Novocastra Labs, Newcastle-upon-Tyne, UK), Ki-67 (Clone SP6, Labvision NeoMarkers, Fremont, CA), and HER2/neu 1 (rabbit polyclonal, DAKO Corporation, Carpinteria, CA). The basic staining procedure used an avidin-biotin-alkaline phosphatase method, modified for antigen retrieval from paraffin-embedded tissue using the procedure of Shi et al [36]. Following overnight incubation with the primary antibodies at 4°C, tissue sections were sequentially incubated with a biotinylated secondary antibody and a streptavidin-alkaline phosphatase conjugate at 37°C for 20 minutes, respectively, (Biogenex, San Ramon, CA, USA) and then visualized using the chromogen/substrate Vector Red (Vector Laboratories, Burlingame, CA, USA). Sections were counterstained with Mayer's hematoxylin, dehydrated, cleared through p-Xylene, and coverslipped. Appropriate positive and negative controls were included for each antibody. During staining, about 7–9% of the samples fell off the slides. The number was similar across epithelial markers, but twice as many benign as malignant samples were missing.

When a trained pathologist (JMC) evaluated all stained specimens to confirm their malignancy status and the presence of epithelial tissue, 118 breast tissue samples were re-categorized as benign or malignant, i.e., the core had been taken from a malignant part of the block although the intent had been to get a benign sample or vice versa. Another 409–433 (depending on the marker) core sections with equivocal features, e.g., connective tissue or fat tissue only or bad dye quality, were excluded. As a result, at least one benign tissue sample was available for 159 women (mean = 1.7 specimens per woman). Quantification of staining was done on individual TMA core sections at a magnification of 20×, using a Nikon Labophot 2 microscope, a 3 megapixel digital camera (Infinity 2–3, Lumenera Inc., Ottawa, Ontario), and color imaging software (Image Pro Plus, Media Cybernetics, Bethesda, MD). The area of all nuclei in the core section was measured by a color selection corresponding to hematoxylin (A). The area of positively immunostained tissue was measured by a color selection corresponding to the Vector Red chromogen (B). For the nuclear stains used in this study, the percentage of staining was expressed as B/A × 100; results were averaged for subjects with several cores.

### Mammographic density assessment

Breast density readings used for the present study had all been obtained previously as part of the NCC study [33]. All mammographic films were scanned with a Kodak LS85 Film Digitizer (absorbance range, 0.001–4.1; Eastman Kodak Company, Rochester, New York) at a resolution of 98 pixels per inch. One of the authors (GM) performed computer-assisted density assessment with the Cumulus package [37]. After the reader determined a threshold for the edge of the breast and the dense tissue, the computer computed the number of pixels that constituted the total and the dense area and the ratio between the two, i.e., percent breast density. To convert the pixels of the area into cm^2^, a factor of 0.000676 was used. The intraclass correlation coefficients (ICC) to assess reliability were 0.96 (95% confidence interval (CI): 0.95, 0.97) and 0.996 (95% CI: 0.995, 0.997) for the size of the dense and the total breast area, respectively. This resulted in an ICC of 0.974 for percent density (95% CI: 0.968, 0.978). For the present study, the cranial caudal view closest to, but before, breast cancer diagnosis was selected; the mean time between the two dates was 10.0 ± 14.8 months.

### Statistical analysis

SAS statistical software package version 9.1 was used for all analyses (SAS Institute Inc., Cary, NC). The dense breast area was square root transformed to normalize the distribution. For ease of interpretation, back transformed mean values are presented. For all histology markers, the mean percentage of stained cells of all available cores per sample was calculated. To assess marker agreement by subject, ICCs were computed. For all six markers, the distributions of samples were skewed with strong left tails. The interquartile ranges were 0.0–8.1%, 0.0–13.8%, 0.0–1.5%, 0.0–14.5%, 0.0–0.6%, 0.8–16.2% for ERα, ERβ, PR, HER2, Ki-67, and PCNA, respectively. Therefore, samples were divided into two categories; negative staining (<10% of cells stained) and positive staining (≥ 10% of cells). Because the number of women with positive staining for PR and Ki-67 was very low (4 and 13 respectively), we dichotomized the results into no *vs*. any epithelial staining. Linear regression models were used to analyze the association between histological markers as categorical variables and mammographic density (absolute density and percent density) as continuous variables. Associations were adjusted for variables previously shown to be associated with mammographic density including age at mammogram, body mass index (BMI), ethnicity, age at menarche, parity, age at first live birth, HT use at mammogram, menopausal status, and family history of breast cancer. Separate analyses stratified by ethnicity (Caucasian and Japanese) and by total size of the breast with the median as cutpoint were also performed.

## Results

Women in the TMA study were slightly younger, were more likely premenopausal, and had a lower BMI than in the original NCC study, but were otherwise similar (Table 1). The study population included 49 Caucasians, 70 Japanese, 21 Native Hawaiians, and 19 women of other ethnicities. Mean total breast area as measured on the mammograms was largest for Caucasian women and smallest for Japanese women (135 and 91 cm^2^, respectively). BMI and total breast area were strongly correlated 0.52 (p < 0.0001). Absolute mammographic density was highest in Caucasian women and in the subgroup of women with other ethnicities (43 cm^2^) and lower in Hawaiian (36 cm^2^) and Japanese women (34 cm^2^). Percent mammographic density was highest among the subgroup of women with other ethnicities (50%) and lowest in Hawaiian women (30%), while it was intermediate in Japanese (40%) and Caucasians (38%).

**Table 1:** Characteristics of women recruited for the TMA study and the original study*

Variable	Original study	TMA study
Sample size (N)	607	159
Ethnicity (%)		
Caucasian	185 (30.5)	49 (30.8)
Hawaiian	80 (13.2)	21 (13.2)
Japanese	287 (47.3)	70 (44.0)
Other	55 (9.1)	19 (12.0)
Age at mammogram	62.1 ± 8.5	59.8 ± 8.7
Body mass index (m/kg^2^)	25.1 ± 5.1	24.4 ± 4.3
Family history of breast cancer (%)	104 (17.1)	17 (10.7)
Age at menarche (%)		
< 13 years	324 (54.4)	86 (54.1)
13–14 years	217 (36.4)	58 (36.5)
>14 years	55 (9.2)	15 (9.4)
Number of children (%)		
0–1	172 (28.3)	43 (27.0)
2 to 3	312 (51.4)	86 (54.1)
>3	123 (20.3)	30 (18.9)
Age at first live birth (%)		
<21 years	80 (13.6)	21 (13.2)
21–30 years	359 (61.0)	102 (64.2)
>30 years	56 (9.5)	11 (6.9)
N/A	94 (16.0)	25 (15.7)
HT use at mammogram (%)		
No use	264 (43.5)	64 (40.3)
Estrogen only	174 (28.7)	46 (28.9)
Estrogen plus progestogen	169 (27.8)	49 (30.8)
Breast measures		
Total breast area	117.9 ± 58.1	110 ± 52.9
Breast density in percent	35.3 ± 23.3	38.4 ± 24.8
Absolute density	35.9 ± 27.0	39.5 ± 23.4
Menopausal Status (%)		
Premenopausal	152 (25.0)	60 (37.7)
Postmenopausal	455 (75.0)	99 (62.3)

* Means ± standard deviation unless stated otherwise

A substantial proportion of the samples were in the low or no staining categories (Table 2). The percentages were 88%, 70%, 49%, 67%, 42%, and 60% for ERα, ERβ, PR, HER2, PCNA, and Ki-67, respectively. A similar proportion of Japanese and Caucasian women were in the highest staining category for all markers; none of the differences was statistically significant. The sample size was too small to examine other ethnic groups. Small differences in breast density were seen between staining categories of several markers, but, with two exceptions in the subgroup of Caucasians, none of the results were statistically significant. Percent density was higher in the overall and stratified analyses for subjects with PR staining (all women: 3.4%; Caucasian: 5.8%; Japanese: 3.5%). In women with higher ERβ staining, percent density was higher in the total population and in Caucasians (2.4% and 11.6%) but not in Japanese. No associations of percent density with ERα and HER2/neu were observed. Percent density was somewhat higher in women with Ki-67 staining, both in the total population (3.4%) and in Caucasians (3.8%) and Japanese (4.4%). Positive PCNA staining showed slightly higher percent density in all women and in Japanese but not in Caucasians.

**Table 2:** Marker expression and mammographic density by ethnicity

Marker		All women (n = 159)			Caucasian (n = 49)			Japanese (n = 70)		
		
		Category*			Category			Category		
		1	2	P-value	1	2	P-value	1	2	P-value
ERα	% density^†^	36.9	35.6	0.75	39.8	23.9	0.04	38.8	44.5	0.39
	Dense area^#^	33.7	33.6	0.98	38.2	26.2	0.18	33.8	38.0	0.50
	Number	122	35		38	11		53	16	

ERβ	% density	35.8	38.2	0.49	33.8	45.4	0.05	40.2	39.1	0.85
	Dense area	33.0	34.6	0.66	34.4	40.9	0.38	35.2	33.3	0.74
	Number	110	47		36	13		47	21	

PR	% density	34.9	38.3	0.28	35.3	41.0	0.30	37.5	41.0	0.57
	Dense area	30.6	36.4	0.09	31.1	45.9	0.03	35.2	34.4	0.90
	Number	77	81		23	25		35	35	

HER2/neu	% density	37.1	36.3	0.82	35.7	40.9	0.38	41.3	37.9	0.57
	Dense area	34.4	33.2	0.74	36.4	36.5	0.99	35.5	33.6	0.73
	Number	104	52		34	15		45	23	

Ki-67	% density	34.3	37.7	0.32	35.5	39.3	0.53	36.8	41.2	0.46
	Dense area	32.9	33.5	0.87	38.7	34.9	0.60	32.8	35.3	0.66
	Number	65	90		16	32		31	39	

PCNA	% density	36.0	38.2	0.50	37.7	37.1	0.91	38.7	42.2	0.52
	Dense area	33.4	33.7	0.94	36.7	36.4	0.97	35.1	33.9	0.82
	Number	95	63		28	20		42	28	

* Categories for ERα, ERβ, HER2/neu and PCNA; category 1 <10% staining, category 2 ≥ 10% staining. Categories for PR and Ki-67; category 1 = no staining, category 2 = any staining

^† ^Mean percent breast density

^# ^Mean dense area in cm^2^

Mean values and p-values calculated using general linear models adjusted for age at mammogram, BMI, ethnicity, HT use at mammogram, menopausal status, parity, age at first live birth, age at menarche and family history of breast cancer.

(ERα = estrogen receptor alpha; ERβ = estrogen receptor beta; PR = progesterone receptor; HER2/neu = Human Epidermal growth factor receptor2; PCNA = Proliferating Cell Nuclear Antigen; BMI = body mass index)

As an exploratory analysis, we stratified by total breast area to capture possible effects due to high adiposity. More women with large breasts had PR expression than women with small breasts (58% vs. 44%, *p *= 0.08) (Table 3). The opposite was seen for ER expression (ERα: 62% *vs*. 77%, *p *= 0.44; ERβ: 56% *vs*. 65%, *p *= 0.30). Women with large breasts and positive staining for all markers, except Ki-67, had higher percent densities, especially for PR (6.2%), ERβ (6.4%), and PCNA (4%). Although not statistically significant, women with small breasts who stained for hormonal markers showed slightly lower percent density except for PR with 4.3% higher density in category 2. Those with positive Ki-67 staining had 4.4% higher density, whereas positive staining for PCNA made no difference among women with small breasts. With few exceptions, the associations with absolute area were similar to the findings with percent density. Restricting the analyses to postmenopausal women did not change the results; no significant associations were observed (data not shown).

**Table 3:** Marker expression and mammographic density stratified by total breast size

Marker		Small total breast area			Large total breast area		
		
		Category*			Category		
		1	2	P-value	1	2	P-value
ERα	% density^†^	41.3	38.0	0.55	30.7	33.7	0.61
	Dense area^#^	29.5	29.1	0.92	34.6	39.7	0.53
	Number	63	16		59	19	

ERβ	% density	41.3	39.5	0.71	29.9	36.3	0.24
	Dense area	28.9	29.7	0.82	34.1	41.2	0.34
	Number	50	29		60	18	

PR	% density	39.0	43.3	0.36	27.1	33.3	0.19
	Dense area	28.8	29.8	0.78	28.3	40.0	0.05
	Number	45	36		32	45	

HER2/neu	% density	41.7	39.8	0.71	30.4	33.5	0.51
	Dense area	30.3	28.6	0.65	35.2	37.6	0.71
	Number	55	24		49	28	

Ki-67	% density	38.2	42.6	0.35	29.7	30.9	0.81
	Dense area	25.4	31.8	0.07	35.8	34.5	0.85
	Number	30	49		35	41	

PCNA	% density	41.0	39.9	0.81	29.9	33.9	0.43
	Dense area	29.4	28.8	0.86	33.7	40.5	0.32
	Number	43	38		52	25	

* Categories for ERα, ERβ, HER2/neu and PCNA; category 1 <10% staining, category 2 ≥ 10% staining. Categories for PR and Ki-67; category 1 = no staining, category 2 = any staining

^† ^Mean percent breast density

^# ^Mean dense area in cm^2^

Mean values and p-values calculated using general linear models adjusted for age at mammogram, BMI, ethnicity, HT use at mammogram, menopausal status, parity, age at first live birth, age at menarche and family history of breast cancer.

(ERα = estrogen receptor alpha; ERβ = estrogen receptor beta; PR = progesterone receptor; HER2/neu = Human Epidermal growth factor receptor2; PCNA = Proliferating Cell Nuclear Antigen; BMI = body mass index)

## Discussion

This investigation of breast density and immunohistochemical marker expression in TMAs observed no significant associations in the entire study population, but it appeared that mammographic density was slightly higher for women with PR expression as compared to those with no PR expression. This observation was consistent across the two major ethnic groups and women with different breast sizes. The difference between low and high categories was 3–4% in density which, if a true finding, may translate into a 6–8% higher breast cancer risk [33]. Only in women with large breasts, mammographic density was slightly higher in subjects with ERα, ERβ, and HER2 expression, but again, the results were not statistically significant. For category 2 expression of Ki-67 and PCNA, percent breast density was slightly higher in the entire population. The findings in Caucasians who, on average, have larger breasts than Japanese largely reflected the results in the subgroup of women with large breasts, whereas the findings in Japanese women tended to be closer to the results of women with small breasts. The higher percent density among women with Japanese ancestry despite their lower breast cancer risk was also observed in the original study [33]. It is due to the small breast sizes that result in a higher percent of the breast occupied by dense tissue. As shown in cross-sectional comparisons, the size of the dense area appears to be a better indicator of risk when different ethnic groups are compared [38,39].

Six previous reports examined the underlying histological markers for breast density; one study used forensic breast samples [11], one examined reduction mammoplasty samples [5], one collected fine needle biopsies [13], two used tissues surrounding non-cancerous breast lesions [9,12], and another one identified non-cancerous tissue from mastectomy specimens [14]. Of these six studies, only one study assessed ERα and PR expression and did not find an association, but the sample size was only 56 [14]. Three studies looked at Ki-67 expression; one found no association with mammographic density [13], one found a positive association [14], and one described less proliferation in dense areas [5]. As far as we know, no previous results for ERβ, HER2/neu, and PCNA have been reported.

The observation that in women with small breasts, percent density was slightly lower for those with higher ER expression, whereas it was higher in women with large breasts and positive ER expression suggests that a possible effect of ER expression on breast density, if it exists, may depend on the amount of local estrogens. Apart from ovarian production, estrogens are metabolized from androgens in adipose tissue [40]. Thus, in women with large breasts, tissue estrogen levels would also be higher due to the larger amount of fat tissue. This idea agrees with a report that women with a nipple aspirate fluid (NAF) phenotype characterized by higher BMI and percentage body fat had higher NAF estrogen levels [41]. Therefore, the higher PR expression in women with large breasts as compared to women with small breasts may reflect responsiveness to estrogen [29]. Despite the non-significant results, these observations may indicate that higher ER expression in combination with high tissue levels of estrogens influence breast density.

This epidemiologic investigation took advantage of the TMA approach that allows assessment of marker expression in a large number of pathologic samples under similar staining conditions [34,42]. Another benefit of TMAs was that multiple markers could be analyzed using sections of the TMA block without exhausting the material. However, a disadvantage of the method is the loss of samples during immunostaining due to lack of adhesion to glass slides and cross-sectional variation in the amount of epithelial tissue. The use of cores with a larger diameter may alleviate that problem. Alternatively, immunohistochemistry could be performed on full sections, if they are available. Another issue with TMAs is that consecutive sections may reveal different types of tissue for the same core due to heterogeneity with tissue depth. As shown before, benign tissue samples were more affected than malignant cores [42,43]. Since a maximum of 4 benign core sections per woman was placed, information on a substantial number of women was obtained. Despite identifying benign specimens for only 159 out of 279 subjects due to the difficulties with benign tissue in the tumor blocks, little evidence for selection bias was detected (Table 1). Obviously benign biopsies would be the preferred source of material for this type of research because benign tissue adjacent to tumor tissue may be different from benign tissue in the other breast or in breast tissue of women without cancer. Unfortunately, that type of tissue is not easy to obtain. The amount of material available from stereotactic biopsies is often too small for TMA preparation and women with benign biopsies cannot be identified through tumor registries. Although breast reduction surgery would yield large amounts of tissue, women undergoing that procedure represent a selected subgroup with predominantly fatty breast tissue and different mammographic patterns [44].

## Conclusion

This investigation illustrates the problems of obtaining benign breast tissue samples for a representative sample of participants in epidemiologic studies. To benefit from the advantages of the TMA technique in future research, it is recommended to select the areas for taking the tissue cores carefully and to minimize the loss of specimens during immunostaining. Based on our few suggestive findings, future mammographic density investigations may pursue estrogenic effects as assessed by PR and cell proliferation using Ki67 and other such markers to examine the question of local estrogen activity in relation to breast density. The slightly higher breast density with PR expression suggests a stronger estrogenic response in mammographically dense breasts possibly leading to stronger cell proliferation, an idea supported by associations of breast density with hyperplasia and other benign breast pathology [8,10,45,46]. Although not statistically significant, the higher breast density with steroid receptor expression in women with large breasts as compared to women with small breasts indicates a role of locally produced estrogens in women with more adipose tissue [40]. On the other hand, it is most likely that chance was responsible for the small differences observed in this exploratory study. Given the weak associations in the current investigations and the discrepant findings across studies, the roles of growth factors and the properties of the stromal matrix in shaping breast density also deserve further attention [4,9,12].

## List of abbreviations

BMI: Body mass index; CI: Confidence interval; ER: Estrogen receptor; HT: Hormone therapy; HTR: Hawaii tumor registry; ICC: Intraclass correlation coefficient; MEC: Multiethnic cohort; NAF: Nipple aspirate fluid; NCC: Nested case control study; PCNA: Proliferating Cell Nuclear Antigen; PR: Progesterone receptor; TMA: Tissue microarray.

## Competing interests

The authors declare that they have no competing interests.

## Authors' contributions

MV carried out the statistical analysis and drafted the manuscript. GM conceived of the study, obtained funding, and directed the statistical analysis. EE participated in the design and the data collection and contributed to the statistical analysis. JSS contributed to the data collection and the statistical analysis. JK evaluated and selected the pathologic specimens and provided input on pathologic issues. BH provided access to the pathologic specimens and contributed to the study design. JMC performed the pathologic evaluation of the TMAs and contributed to the study design. All authors read and approved the final manuscript.

## Pre-publication history

The pre-publication history for this paper can be accessed here:

http://www.biomedcentral.com/1471-2407/9/182/prepub
